# The Influence of Individual and Team Cognitive Ability on Operators’ Task and Safety Performance: A Multilevel Field Study in Nuclear Power Plants

**DOI:** 10.1371/journal.pone.0084528

**Published:** 2013-12-31

**Authors:** Jingyu Zhang, Yongjuan Li, Changxu Wu

**Affiliations:** Institute of Psychology, Chinese Academy of Sciences, Beijing, China; Institut Pluridisciplinaire Hubert Curien, France

## Abstract

While much research has investigated the predictors of operators’ performance such as personality, attitudes and motivation in high-risk industries, its cognitive antecedents and boundary conditions have not been fully investigated. Based on a multilevel investigation of 312 nuclear power plant main control room operators from 50 shift teams, the present study investigated how general mental ability (GMA) at both individual and team level can influence task and safety performance. At the individual level, operators’ GMA was predictive of their task and safety performance and this trend became more significant as they accumulated more experience. At the team level, we found team GMA had positive influences on all three performance criteria. However, we also found a “big-fish-little-pond” effect insofar as team GMA had a relatively smaller effect and inhibited the contribution of individual GMA to workers’ extra-role behaviors (safety participation) compared to its clear beneficial influence on in-role behaviors (task performance and safety compliance). The possible mechanisms related to learning and social comparison processes are discussed.

## Introduction

The performance of main control room (MCR) operators is key to the efficiency and safety of high risk process control industries such as nuclear power plants (NPPs) [1]. To maximize their performance, researchers and practitioners need to find a reliable predictor of operators’ performance in real work environments [2]–[5]. Among many individual difference predictors of work performance, general mental ability (GMA) might be one of the most important [6], especially for performing complex tasks such as controlling a nuclear reactor [7].

However, several important issues still require attention. In the first place, although some laboratory studies have investigated the influence of GMA on process control operators’ performance [3], [8], the lack of field studies restricts the ecological validity of GMA research in this industry. Secondly, compared with workers’ primary task performance (e.g. efficiency and accuracy in fault finding and operation), less research has been conducted on the influence of GMA on safety performance (e.g. complying with rules and helping others to behave safely) which is also highly valued in this industry [9]. While these aspects of performance have been mostly researched in the framework of motivation, social influence and stress [10], they may also be influenced by GMA for two reasons. First, according to the job-demand-control model [11], when operators have equal job-demands, high GMA ones are more likely to take control of their work than their low GMA counterparts and therefore have better safety performance [12], [13]. Second, from a learning perspective, GMA and work experience can interact to influence one’s job-related knowledge, which can in turn promote both task and safety performance [9], [14]. Moreover, as control room operators generally work in teams, team members’ ability can compensate or hinder individual’s capability to perform effectively and safely [15]–[17]. When team members’ GMA levels are all very high, additional cognitive resources can be used to reduce potential risks [16]. However, having too many high GMA members in one team may produce some negative impacts known in the social comparison literature as the “big-fish-little-pond effect” (individual’s self-evaluation and performance can be influenced by their relative competence compared with their schoolmates, colleagues, etc.) [18], [19]. In this sense, it is intriguing to ask whether the GMA composition of the whole team (team GMA) might provide more information than only using the individual level GMA as a performance predictor in field NPP settings.

With these unresolved questions in mind, the current study was conducted to establish an overall picture of the GMA-performance relationship under real life conditions. Using a sample including almost all licensed main control room operators presently working in China’s nuclear power industry (312 operators from 50 shift teams), this study sought to investigate the joint influence of individual GMA, team GMA and work experience on both task and safety performance among this special category of workers.

### Individual GMA and Task/Safety Performance

General mental ability, also known as general intelligence or the g factor, can be best described as the general capability for processing complex information in everyday life from problem solving to social adaptation [20], [21]. Operationally, it is measured by some particular GMA tests (e.g. Raven’s Progressive Matrices Test) or batteries including different cognitive tests (e.g. General Aptitude Test Battery) [22]. Many laypersons, and even human resource practitioners and trained psychologists, tend to think GMA can only predict academic performance rather than work outcomes, which may be influenced by other specific abilities or personality variables such as conscientiousness [23]. However, numerous studies have found that GMA is the best single predictor of work performance or income [6], [14], [20], [22]. According to some recent meta-analyses, the criterion related validity is as high as.51 for GMA as compared to.23 for consciousness, .23 for core-self-evaluation and.22 for emotional intelligence [6], [24], [25]. Further, the level of task complexity has been found to be an important moderator of the GMA-performance relationship: the more complex the job, the higher the GMA-performance correlation [20]. In this sense, the work performance of main control room operators in nuclear power plants – a typical highly complex job – should be positively influenced by operators’ GMA levels.

However, it is important to note that most of these findings are based on task performance, namely, the efficiency and accuracy of performing one’s formally required tasks. In an NPP context, these tasks include monitoring the system, recognizing warning signals, finding certain system faults and choosing the best strategies to solve these problems. While knowledge-based mental processes are highly involved [3], [7], it is not surprising that this dimension of performance can be highly influenced by one’s general mental ability.

Safety performance, including safety compliance (in-role activities to promote safety such as following certain rules and regulations) and safety participation (voluntary extra-role behaviors such as helping others or making suggestions to the whole organization to promote safety), has been proved to be the most direct antecedent of accidents and injuries [26]. Unlike task performance, safety performance is generally considered to be routinized and non-innovative behavior which requires high motivation and self-regulation rather than problem-solving abilities [9], [27], [28]. Therefore, GMA may not have significant influence on these “simple” behaviors. Nevertheless, evidence shows that high GMA is related to a reduced involvement in accidents and more organizational participatory behaviors [29]–[31]. These findings can be explained in a job-demand-control framework [11]: as GMA is one of the most important abilities required by MCR operators [12], those with a high level of GMA are more likely to take control of their work given job-demand being equal, which can in turn increase their safety performance [13]. As a result, we postulate Hypothesis 1:

H1: individual GMA will be positively related to both task performance and safety performance.

### The Joint Effect of GMA and Work Experience

However, the influence of GMA on performance may depend on worker’s experience, particularly as a learning process is highly involved. To note, GMA is not “the amount of information people know”, but “the ability to recognize, acquire, organize, update, select, and apply it effectively” ([20], p. 93). In this way, individuals with high GMA may not be natural high level performers, but they are able to acquire job-related knowledge and skills at a faster pace and to a higher level than others, which can in turn improve both their task and safety performance [6], [9].

To illustrate the learning process, three possible temporal patterns have been proposed, taking work experience into consideration. The first convergent view suggests that while individuals with high GMA may learn faster at the beginning of their work, as more experience is acquired, all individuals may obtain enough knowledge to perform their tasks. As a result, individuals with both high and low GMA will ultimately have the same level of performance. This view is based on the premise that the skills and knowledge required to perform one’s job are finite and can be reached in a limited time. In contrast, the divergent view suggests that the work-related knowledge one needs to learn is far more complex than generally expected, even in the least demanding jobs [14]. As a result, individuals with high GMA can not only learn faster but also reach a higher knowledge level than their lower GMA counterparts, so their performance difference will become larger as more experience is accumulated. Between these two positions, there is a third, parallel view suggesting the difference will remain the same as experience increases.

However, empirical research on task performance has disproved the convergent view [14], [32]–[34], though evidence is inconsistent regarding the other two views. An early study [6], using a restricted range of work experience (<7 years), found supportive evidence for the parallel view that the correlations between GMA and task performance were positive and invariant across different experience groups. But, by including more experienced workers (10+ years), a later study confirmed the divergent view to be more likely [33]. In a recent study, Judge et al. used a twenty year longitudinal design to investigate the effect of GMA on variables related to career success (e.g. income) [32]. Besides finding a significant positive effect of GMA on people’s initial career success, they also noted that while high GMA individuals continuously improved their conditions throughout their life, the career of low GMA individuals declined after they reached a plateau in the middle of their career. Taken together, the accumulating evidence generally supports the divergent view.

Although no research, to our knowledge, has investigated the joint effect of GMA and experience on safety performance, the divergent pattern may also occur in safety performance since safety-related knowledge has been found to be an important predictor of safety performance [9], which may also be acquired faster by those with high GMA, so we postulate Hypothesis 2:

H2: GMA will interact with work experience to influence task performance and safety performance and show a divergent pattern.

### Team GMA and Performance: Cooperation and Social Comparison

Beyond the abovementioned individual level influences, team composition (e.g. team GMA) can also influence individual operators’ performance since most of their tasks are accomplished through teamwork. Team GMA, referring to the team’s overall ability and cognitive resources to process information, is generally operationalized as the mean score of all team members’ GMA test performances [15], [16], [35]. Empirically, it has been found that team GMA is linked with more accurate shared mental models [36], faster learning of task related knowledge [37], and better long-term cooperation [38]. Since all these benefits can be translated into task and safety performance, the relationship between team GMA and both kinds of performance may be positive.

Although these greater cognitive resources could help the team to perform complex tasks, in a team full of high-ability individuals, some members may nevertheless feel less valued which may in turn undermine their performance. According to social comparison theory, workers inevitably compare themselves (especially their abilities) with similar others at the workplace (e.g. their teammates) to form self-evaluations such as whether they can perform tasks well or can be accepted by the whole team [18]. While downward comparison (with those who are less capable), may increase their confidence and job satisfaction [39], [40], upward comparison may have negative influences on their self-concept and cooperative behaviors [39], [41]. Although no research, to our knowledge, has linked these processes with either individual or team GMA composition in organizational settings, research in educational psychology has found a prevalent “big-fish-little-pond” effect: though individual ability has a positive influence on students’ academic confidence, higher average ability of schools can cast a negative influence [19]. The reason is that when individuals are imbedded in an environment composed of highly competent individuals, the unavoidable upward comparison can seriously undermine a student’s self-concept as a capable learner. The aphorism “better to be a big fish in a little pond than a small fish in a big pond” grasps some aspects of this effect. In an organizational context, the reduced self-concept, job satisfaction and increased rivalry resulting from the fish-pond effect are more likely to influence workers’ cooperative and participatory behaviors (extra-role behaviors) rather than their in-role work behavior [42], [43]. As a result, compared with task performance or safety compliance behaviors, safety participation is more likely to be influenced by this effect. Taken together, while team GMA may increase task performance and safety compliance behavior, it should have a mixed effect on safety participation behavior. As a result, we postulate Hypothesis 3:

H3: team GMA will be positively related to both task performance and safety compliance (H3-a) but not to safety participation (H3-b).

This effect could, moreover, be larger for those individuals with high GMA. As they may have been the best in their schools and/or in other teams, if they enter a team with highly competent colleagues, they may feel like “a big fish in a little pond suddenly turned median or small when thrown into a big pond with many big or bigger fish” ([44], p. 11). Consequently, their confidence and motivation to help could be severely compromised. For example, in research on a gifted education program, it was found that when students with very high GMA were selected to receive training together, their academic confidence was significantly reduced [45]. However, for those with low GMA, this effect may have relatively little influence. As a result, while individual GMA may have a positive influence on safety participation behavior in teams with low average GMA, in those groups with high average GMA, it may have no or negative influences on such behaviors. On the other hand, this effect may also have little to do with in-role task related behaviors. Thus we postulate Hypothesis 4:

H4: team GMA will not influence the effect of individual GMA on task performance and safety compliance (H4-a), but will reduce the effect of individual GMA on safety participation (H4-b).

### Overview of the Current Study

To test the hypotheses we have proposed, we conducted a field study by investigating nearly all incumbent control room operators in all running nuclear power plants in China. This sample was large enough at both individual (312 operators) and team (50 teams) level to provide important data about the influences of individual GMA, team GMA and work experience on both task and safety performance of process control operators.

## Methods

### Ethics Statement

The study and its consent procedure were approved by the Institute of Psychology, Chinese Academy of Sciences. With the help of the Research Institute of Nuclear Power Operation (China) responsible for the operator licensing process in this country, we gained permission from plant managers to conduct our research with an agreement that the original data should not be disclosed to a third party. We surveyed and tested operators during the teams’ regular training sessions. They received verbal informed consent that their individual responses would be analyzed in an anonymous manner. This consent was also explicitly printed on the first page of the survey which was collected unsigned afterwards to document this process. Thereafter, their GMA was tested and their demographic information surveyed. Their performance ratings (task performance, safety compliance and safety participation) were collected later from the team supervisors.

### Participants

In total, 345 main control room operators from two major nuclear energy corporations in mainland China agreed to participate in this research. After excluding 33 members from 5 teams whose supervisor rated performance was not successfully collected, 312 valid responses from 50 shift teams were used for the final data analysis. All participants were male between 20 and 40 years old (M = 29.5, SD = 2.48). Team sizes were between 3 and 10 (M = 6.24, SD = 2.18). In general, a minimum functional team consists of a reactor operator, a turbine operator and an auxiliary operator. However, unlike the virtual and temporary teams frequently adopted in laboratory studies which generally have 3–5 members, larger groups are common in real organizational settings, in order to train new operators or provide enough redundancy to guarantee safety.

### Measurement

#### 1. Work experience

Work experience was collected through self-report by asking how many years the operators had worked in their current positions (as a control room operator). It ranged from 2 to 15 years (M = 6.64, SD = 2.63).

#### 2. General mental ability

The operators completed the Raven’s Standard Progressive Matrices (SPM) during their regular training sessions. As one of the most widely used nonverbal tests of GMA, the SPM has 60 diagrammatic puzzles exhibiting serial changes in two dimensions simultaneously which participants need to examine in order to find the missing part of each puzzle among the provided options [46], [47]. The SPM has good reliability and validity across different groups with varying age, cultures and health conditions [46]. For the current research, testing was conducted in groups with a 40-minute time limit. We transformed the raw scores into standard scores based on the test manual [46] and the Chinese norms [48] in order to make it comparable across different age groups. The average score of our sample was 111.17 (SD = 10.53).

#### 3. Task and safety performance

Task performance was measured by the 7-item general task performance scale [49], whereas safety compliance and participation were measured by two three-item scales [26]. The Chinese versions of the two scales have been validated in previous research [4]. In order to avoid common factor variance, supervisor rating was used in the current study. The supervisors were asked to rate the performance of their subordinates’ behavior in the past three months on a 5-point Likert scale from 1 “almost never” to 5 “almost always”. Sample items were “[he] adequately completes assigned duties” (task performance), “[he] uses all necessary safety protection in work” (safety compliance) and “[he] voluntarily carries out tasks or activities that help to improve workplace safety” (safety participation).

Confirmatory factor analysis suggested that the three-factor model (df = 61, χ^2^ = 182.4, NFI = .91, CFI = .94, RMSEA = .08) was better than the single factor solution (df = 64, χ^2^ = 431.8, NFI = .77, CFI = .81, RMSEA = .14) and any two factor models (the best two factor model was the one in which three items measuring safety participation loaded on one factor and the other 10 items on another: df = 63, χ^2^ = 246.2, NFI = .88, CFI = .91, RMSEA = .10). The Cronbach’s alpha coefficients for task performance, safety compliance and safety participation were.81, .73 and.87, respectively. To note, as the 6th and 7th items of task performance were reversed questions, we set their error terms to be correlated in order to improve the overall fit of the measurement model in the confirmatory factor analysis.

#### 4. Control variables

To control for any difference between the two energy corporations, a dummy variable named Organization was created for further analysis (where 0 and 1 represent the two organizations, respectively). As age and work experience were highly correlated in the current sample (r = .81), to avoid collinearity, we used entering age to control for other non-cognitive changes related to age. Entering age was produced by subtracting the work experience from their current age. It has lower correlation (r = −.39) with work experience. Group size was also controlled for as it has been found to be an important factor in predicting the performance of process control operators [50], [51].

### Data Analysis

The data were analyzed anonymously to guarantee the privacy of all participants. To test the hypotheses with a multilevel data structure (operators nested in teams), we adopted the method of hierarchical linear modeling (HLM, [52]). First, a null model with no predictors at both level-1 (individual level) and level-2 (team level) was conducted to quantify the within-team and between-team components of variance for each performance criterion.

Next, we tested our hypotheses in 3 steps. Firstly, to test H1, model 1 was constructed where team size and organizational affiliation were entered at level 2 (all grand-mean centered) as control variables, while individual’s entering age (as a control variable) and GMA (centered on group mean) were entered at level 1. Secondly, model 2 was constructed to test H2. Since work experience is a temporal variable and its absolute contribution was of interest (i.e. how many years after entering the organization can differentiate the performance between high and low GMA operators), it was centered on grand mean and entered into level 1 [53]. In addition, the interaction terms between work experience and GMA were entered at level 1 on the basis of model 1. Thirdly, model 3 was constructed to test H3 and H4: team level GMA (created by averaging all team members’ GMA scores) was entered at level 2 (grand mean centered) to predict both the intercepts and the coefficients of individual GMA on three performance criteria.

The models were depicted as follows:

Model 1: Y*_ij_* = *γ*_00_+ *γ*_01_ ⋅ Organization+*γ*_02_ ⋅ Size +*γ*_10_ ⋅ E-Age+*γ*_20_ ⋅ GMA_ind_+*u_0j_*+*e_ij_*

Model 2: Model 1+ *γ*_30_ ⋅ Experience+ *γ*_40_ ⋅ GMA_ind_ ⋅ Experience

Model 3: Model 2+ *γ*_03_ ⋅ GMA_team_+*γ*_21_ ⋅ GMA_team_ ⋅ GMA_ind_

Y*_ij_* is the performance evaluation of operator *i* in team *j*; *γ*_00_ is the general intercept; *γ*_01_∼*γ*_03_ are the regression coefficients of the second level constructs (organization, team size and team GMA, respectively); *γ*_10_∼*γ*_40_ are the regression coefficients of the individual level constructs (entering age, individual GMA, work experience and the interaction between individual GMA and work experience, respectively); *γ*_21_ is the regression coefficient of the effect of team GMA on the influence of individual GMA on Y*_ij_* (the cross-level interaction); u*_0j_* is the error term on the group level in the intercept, and e*_ij_* is the error term on the individual level.

## Results

### Initial Analysis

Table 1 presents the descriptive statistics and inter-correlations between all variables at individual and team levels. At the individual level, work experience and GMA had positive correlations with all performance dimensions. At the team level, large teams had lower levels of performance. These patterns were consistent with previous research [14], [15], [32]–[36], thus suggesting that the criteria in the current study are generally valid. Organizational affiliation was also treated as a control variable in further analysis.

**Table 1:** Means, standard deviation, and correlations of all variables (N = 312).

	Mean	SD	1	2	3	4	5	6	7
Individual level									
1. Entering Age	22.82	1.57	–						
2. Experience	6.64	2.74	−.39**	–					
3. GMA	111.35	9.05	.02	.07	–				
4. Task Performance	4.54	.43	.01	.18**	.23**	–			
5. Safety Compliance	4.55	.48	_._05	.23**	.25**	.66**	–		
6. Safety Participation	3.92	.79	.02	.22**	.20**	.57**	.61**		
Team level									
7. Organization	.49	.50	−.15*	.05	.04	.13*	.15**	.17**	–
8. Team size	6.24	2.18	−.04	−.32**	−.12*	−.24**	−.19**	−.28**	.28**

Note: *p<.01, **p<.001.

### HLM Results

By calculating the intra-class correlations of the null model, it was found that a significant proportion of total variance was between-teams (task performance = 55.3%, safety compliance = 52.9%, and safety participation = 69.5%). The existence of such large between-team variance justified the use of the multilevel analysis rather than treating them as individual level random errors. Next, we performed the HLM analysis to test the other 3 models. Details are presented in Table 2.

**Table 2:** HLM results predicting task and safety performance of NPP MCR operators.

Parameters	DV = Task performance			DV = Safety compliance			DV = Safety participation		
	M1	M2	M3	M1	M2	M3	M1	M2	M3
Intercept	4.47** (.05)	4.47** (.05)	4.47** (.04)	4.57** (.05)	4.57** (.05)	4.58** (.04)	3.93** (.09)	3.94** (.09)	3.94** (.08)
Individual Level									
E-Age	−.014 (.011)	−.003 (.011)	−.005 (.011)	−.010 (.012)	.007 (.012)	.005(.013)	.007(.017)	.031+(.016)	.027+(.016)
GMA_ind_	.004*(.002)	.005**(.002)	.004+(.002)	.002 (.002)	.005*(.002)	.004+(.002)	.004*(.002)	.006**(.002)	.003 (.003)
Exp		.017+(.009)	.014 (.009)		.027*(.012)	.022+(.012)		.041**(.012)	.038**(.012)
GMA_ind_ × Exp		.002*(.001)	.002*(.001)		.003**(.001)	.003**(.001)		.002*(.001)	.003**(.001)
Team Level									
Organization	.135 (.113)	.138 (.110)	.113 (.102)	.144 (.111)	.152 (.107)	.120 (.090)	.284 (.217)	.284 (.213)	.258 (.206)
Team Size	−.057*(.025)	−.052*(.025)	−.034 (.022)	−.051+(.026)	−.044+(.024)	−.021 (.020)	−.116*(.049)	−.102*(.047)	−.082+(.045)
GMA_team_			.030**(.007)			.040**(.008)			.035+(.017)
Cross-level Interaction									
GMA_team_ × GMA_ind_		−.0002 (.0002)			−.0001 (.0002)			−.0006* (.0002)	
Pseudo R^2^	.05	.07	.17	.04	.10	.26	.07	.10	.13

Note: N = 312 operators (Level 1) in 50 teams (Level 2); NPP: nuclear power plants; MCR: main control room; DV: dependent variable; E-Age: Age at which the operators start working; Organization: organizational membership. Parenthetical values indicate robust standard errors.

p<.10;

p<.05;

p<.01.

Hypothesis 1 predicted that GMA would be positively related to performance. The regression coefficients for GMA (*γ*_20_) in model 1 were significant for task performance (*γ*_20_ = .004, p<.05) and safety participation (*γ*_20_ = .004, p<.05) but not for safety compliance (*γ*_20_ = .002, *ns*). The results indicated that operators with high GMA generally have higher levels of performance.

**Figure 1:**
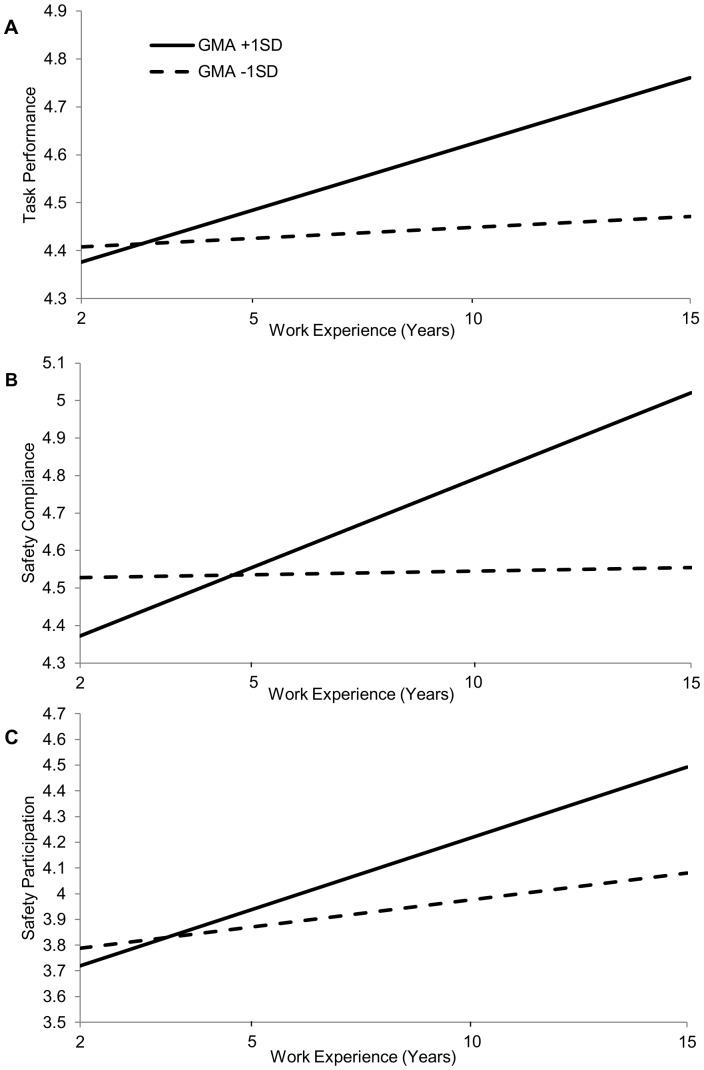
The Joint Effect of Work Experience and Individual General Mental Ability on Task Performance (A), Safety Compliance (B), and Safety Participation (C), Controlling for Team Level Variance.

Hypothesis 2 predicted that GMA would interact with work experience to influence performance (the divergent pattern). The regression coefficients of the interaction terms between GMA and experience were positive and significant for task performance (*γ*_40_ = .002, p<.05), safety compliance (*γ*_40_ = .003, p<.01), and safety participation (*γ*_40_ = .002, p<.05). We further depicted the simple slopes of the interaction in Figure 1 based on the approaches recommended by Preacher, Curran, and Bauer [54]: the difference in performance between individuals with high and low GMA became larger as they both became more experienced (significantly higher after 5 years for task performance and safety compliance, and 6 years for safety participation). These results indicated that the divergent pattern is valid not only for task performance but for safety performance.

Hypothesis 3 predicted that the whole team members’ GMA would have a positive influence on task performance and safety compliance but less so on safety participation. The results indicated that the team mean GMA score had a positive and significant influence on task performance (*γ*_03_ = .030, p<.01), safety compliance (*γ*_03_ = .040, p<.01), and safety participation to a minor degree (*γ*_03_ = .035, p = .08). The results indicated that operators in intelligent teams have better task performance and safety compliance behavior; however their improvement in safety participation behavior is relatively smaller. As a result, H3 is generally confirmed.

**Figure 2:**
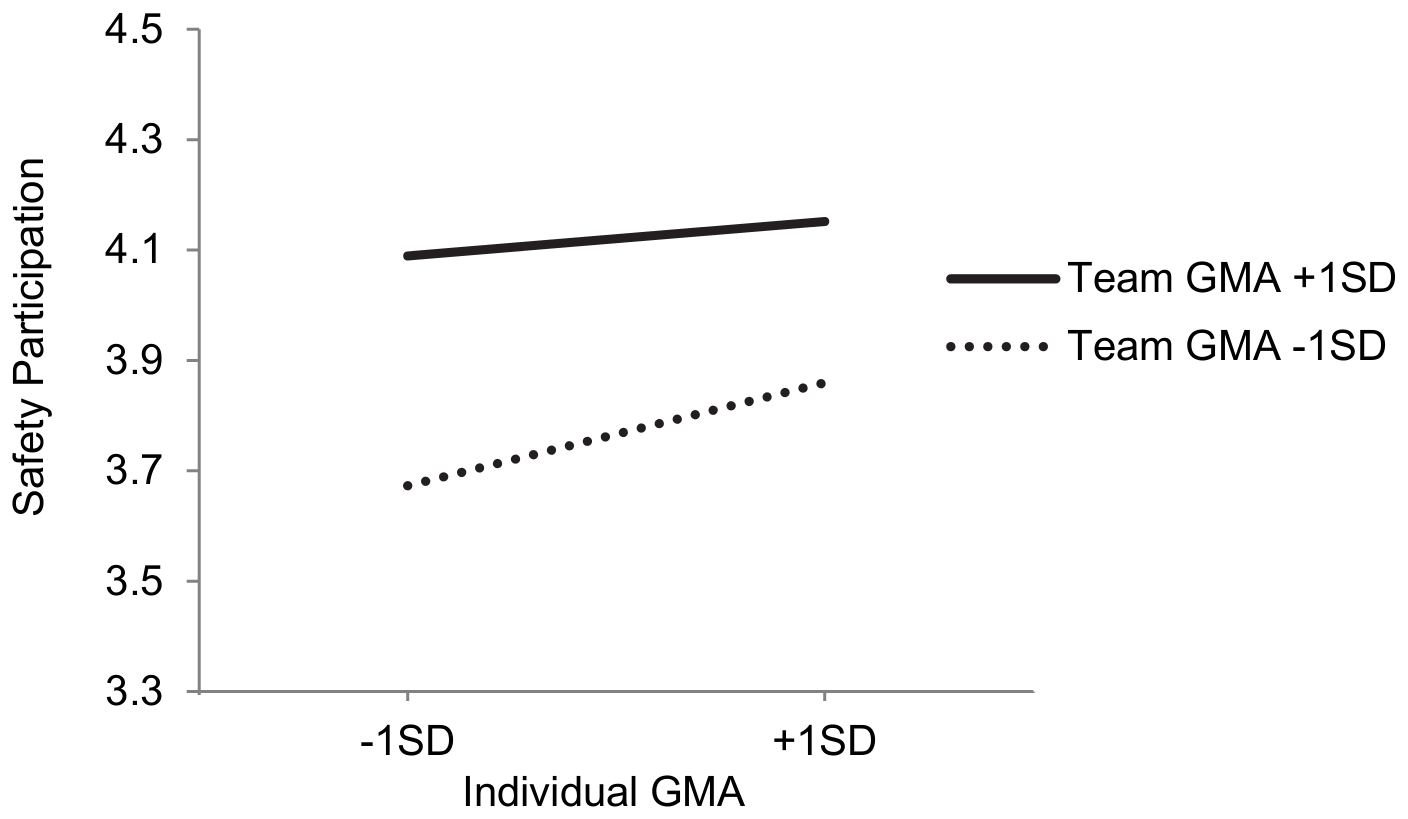
The Joint Effect of Individual GMA and Team GMA on Safety Participation.

Hypothesis 4 predicted that the whole team members’ GMA would reduce the influence of individual GMA on operator safety participation behaviors but not their task performance and safety compliance behaviors. There was a significant cross-level interaction for safety participation (*γ*_21_ = −.0006, p<.05), but not for the other two criteria. Further analysis of simple slopes (Figure 2) suggested that in low GMA teams (one SD below average), individual GMA is significantly related to safety participation (B = .0055, p<.01), however, in average and high GMA teams (one SD above average), individual GMA is not related to such behavior (B = .0032, p = .26 and B = .0008, p = .83, respectively). These results indicated that only in less intelligent teams, helping others to behave safely is influenced by operator’s intelligence (“big-fish-little-pond” effect). On the other hand, individual GMA is always positively related to their task performance and safety compliance regardless of the team GMA level. As a result, H4 is generally confirmed. However, it is worth noting that although we did find the “big-fish-little-pond” effect, Figure 2 shows that the main effect of team GMA is far greater and therefore even a very intelligent operator in a team with low GMA level does not achieve a better performance than operators in a high GMA team.

To note, the same regression analyses performed for both organizations separately produced similar coefficients to those based on the whole sample, except for the effects of team size which were different between the two organizations (y = −.10 to −.22, p<.001, vs. y = −.01 to −.02, p>.15). One possible reason may be that the first organization has recently recruited many novice operators. Therefore, teams allocated with more such operators will have large size and lower performance.

## Discussion

The present study has sought to investigate how individual work experience and team GMA composition interact with individual GMA to influence operators’ performance in a nuclear power plant context. Several findings are worthy of discussion.

Firstly, in replicating previous research that GMA can positively influence operators’ task performance, we also found individual GMA was predictive of safety performance, especially when further considering the joint effect of GMA and work experience. The divergent patterns we found indicated that the difference in performance between those with high and low GMA was not significant until a certain amount of experience had been accumulated (about 5 years for task performance and safety compliance and 6 years for safety participation). This analysis also revealed why GMA did not have a main effect on safety compliance, because in younger groups, individuals with high GMA are even less likely to obey the rules compared to their counterparts with low GMA, though the reverse occurred in older groups. Since individuals with high GMA are generally more self-confident, they may believe they can perform their tasks well without complying with certain rules or regulations every time, but when they acquire enough domain specific knowledge, they may be more able to understand the value of obeying them.

Taken together, this finding shows that cognitive ability is also highly important for conducting safety behaviors. In highly complex working environments such as NPPs, operators have to make a correct judgment of the current situation and decide the most proper sequence of procedures to adopt before taking any actions or making suggestions. While these mental processes are highly influenced by one’s GMA level, the link between GMA and safety performance is often ignored in recent research [9], [10]. Moreover, as the judgment process requires a great deal of declarative and procedural knowledge which does not come from thin air, even those with high GMA are not natural high performers (i.e. they did not perform better when they lacked certain experience). Nevertheless, they may be at an advantage insofar as they are more capable learners. With more experience, they can gain a higher level of both task- and safety-related knowledge which can in turn improve their task and safety performance.

It is interesting to note, while work experience had a positive influence on safety participation even for those with low GMA, its influence on task performance and safety compliance was only positive for high GMA operators. The reason might be that it is more cognitively demanding to learn the knowledge relevant to fulfill in-role requirement rather than extra-role behaviors, so low GMA operators may have slower improvement in their in-role performance, but can still steadily improve their safety participation behaviors.

Secondly, in addition to individual GMA, we also found that team level GMA is positively linked with operators’ task and safety performance (albeit less so on safety participation behaviors). This finding highlights the function of a higher order team level construct that could explain more variance beyond mere individual level analysis. If we consider team GMA as a measure of overall cognitive resources that are possessed by the whole group, high team GMA means more cognitive resources to allocate. According to a human performance model, given the same level of task requirement, the higher the operators’ ability, the more free capacity they have. When the team members have more free capacity in performing their tasks, they are more likely to reflect more thoughtfully, avoid overload and have better mutual monitoring.

Thirdly, the cross-level interaction between team GMA and individual GMA on safety participation behavior was also found. Although the advantage of individual GMA was still evident in task performance and rule compliance behaviors, the difference between high and low GMA operators in safety participation behavior diminished when a team’s average intelligence level was higher. This indicates the presence of the “big-fish-little-pond” effect: when surrounded by equally or more capable team members, high GMA operators do not show more participatory behaviors than their low GMA team members. This might result from reduced motivation as they may think their advice or help will not be valued [39]. This is consistent with previous research that found compared with clearly prescribed in-role behaviors, extra-role citizenship behaviors are more likely to be influenced by attitudinal variables and social comparison processes [41], [43]. It should be noted that, although our findings generally support the notion that social comparison processes may influence organizational safety behaviors, Figure 2 shows that the overall benefit of team GMA outweighs its potential costs on performance. To put it in another way, although the “big-fish-little-pond” effect exists, more intelligent teams maintain a clear advantage, so even a less capable individual from such a team can do better than a highly capable operator from a less intelligent team. This may suggest that the cognitive resources at the team level are more important than the possible motivation reducing effect resulting from the social comparison process. But further research is needed to investigate whether the latter process is more influential on self-evaluation variables such as self-efficacy. If this is the case, there may be more negative influences in the long run than we have observed in the current study.

We also found that team size is negatively related to operators’ performance. This result is inconsistent with a previous finding [51] which, however, only observed teams with three or four operators in laboratory experimental settings. While more team members may reduce each member’s workload in laboratories, in real organizational contexts, larger teams may encounter other problems such as increased complexity of coordination, social loafing and motivation loss, as well as disorder in role structure [50]. However, it is interesting to note that this effect became non-significant when team GMA was entered into the model (model 3). One possibility is that in natural settings, when team members are not performing well, the team leaders often ask for more personnel regardless of their capabilities. In our sample, the negative correlation between team size and GMA (r = −.12, p<.05) partially supports this explanation. However, when taking our findings on the effect of team GMA into consideration, this practice may actually worsen the situation by producing a large but incapable team. This might, in turn, increase the potential risks for the whole organization since in most cases it is unsafe behaviors that increase the likelihood of injuries and accidents. Practitioners should therefore consider new solutions for teams with lower performance: it seems that increasing the average GMA of a team is more beneficial than increasing the team size.

It is important to note that the present study simultaneously investigated three performance criteria. In the first place, it has replicated findings of previous research concerning the influence of GMA on task performance which provides a good basis for further analysis of the two dimensions of safety performance. In general, task performance and safety compliance showed a quite similar pattern whereas safety participation behavior, on the other hand, showed a different pattern as manifested in the interaction between individual GMA and experience as well as the cross-level interaction between team GMA and individual GMA. This is consistent with Neal and Griffith’s theorization that safety compliance is more related to in-role behavior while safety participation more related to extra-role behavior. In addition, the present findings have suggested that these two types of behaviors may have a different dependence on intelligence-knowledge and social comparison processes.

Several methodological strengths and limitations of our study should be mentioned. First, compared to some previous research using self-report measures of safety performance, the present study used supervisor ratings as dependent variables. It is also worth mentioning that the rating bias in our research is likely to be very low as the raters are quite familiar with objective behavior-based performance evaluation which is highly valued and commonly used in an NPP context. As a result, the between-team variance resulting from rating bias (e.g. a leniency or strictness effect) is minimized. Second, by using a multilevel design, the present study was more robust than research that does not control for and quantify team level differences. In fact, the high intra-class correlations suggested that the multilevel analysis was not only proper but necessary. The fact that the between-team difference could be predicted by objectively measured team constructs (size and overall mental ability) suggested that they reflected real differences between groups. Moreover, if some rating bias still exists, it can be statistically accounted for as the between-team residuals, thus making the model more reliable.

On the other hand, it is necessary to recall the cross-sectional nature of the present study. Readers should remain cautious about explaining the findings as totally causal. Although individual GMA is generally considered an invariant psychological property which cannot be determined in reverse by one’s performance, at the team level, the relationship may be more complex. For example, high performance teams may have higher priority and stricter criteria in selecting new members, such that only highly capable candidates may enter these teams. This may cause them to have both a higher performance and higher GMA scores, thus overestimating the relationship between team GMA and performance. To rule out this possible alternative explanation, future studies should track the team changes over a certain time period or explicitly measure their personnel management systems. Longitudinal design may also provide more concrete evidence concerning the learning process. However, due to its cost and natural attrition, such a study is very difficult to implement.

### Implications

The performance of MCR operators is undoubtedly important for its direct influence on the efficient and safe operation of nuclear power plants. However, from both theoretical and practical perspectives, compared with other personality variables such as conscientiousness, the significant impact of GMA (especially its effect on safety performance) has not been adequately appreciated. The present study has provided concrete evidence on the high predictive validity of both individual and team GMA on operators’ task and safety performance. Theoretically speaking, this finding suggests that rather than being driven solely by one’s emotion or motivation, deep-level cognitive processing is highly involved in safety-related behavior. Moreover, the underlying mechanisms of the two dimensions of safety performance (e.g. learning, team process, social comparison) may be different and warrant further research. Practically speaking, as the training cost of a qualified operator is very high, a valid, efficient and long-time predictor of their performance, such as GMA should be considered as a selection tool (or at least an important reference) for human resource practitioners to use in operator selection and team organization. Supervisors should additionally be aware that increasing team size cannot remedy the lack of team GMA and may result in negative influences.
